# The Role of Tumour Stroma in Colorectal Cancer Invasion and Metastasis

**DOI:** 10.3390/cancers3022160

**Published:** 2011-04-26

**Authors:** John Conti, Gareth Thomas

**Affiliations:** Cancer Sciences Division, Southampton University, Somers Building, Southampton General Hospital, Mailpoint 824, Tremona Road, Southampton SO16 6YD, UK

**Keywords:** colorectal cancer, stroma, myofibroblasts, tumour microenvironment

## Abstract

Colorectal cancer (CRC) is a major cause of mortality in western society with a 5-year survival of approximately 50%. Metastasis to the liver and lungs is the principal cause of death and occurs in up to 25% of patients at presentation. Despite advances in available techniques for treating metastases, the majority of patients remain incurable and existing adjuvant therapies such as chemotherapy are only of limited effectiveness. Understanding the molecular mechanisms underlying the metastatic process may allow us to identify those at greatest risk of recurrence and discover new tumour targets to prevent disease progression. It is now apparent that tumour stroma plays an important role in promoting tumour progression. A pronounced desmoplastic reaction was associated with a reduced immune response and has been shown to be an independent poor prognostic indicator in CRC and cancer recurrence. Determining the cause(s) and effect(s) of this stromal response will further our understanding of tumour cell/stromal interactions, and will help us identify prognostic indicators for patients with CRC. This will not only allow us to target our existing treatments more effectively, we also aim to identify novel and more specific therapeutic targets for the treatment of CRC which will add to our current therapeutic options.

## 1. Introduction

Colorectal cancer (CRC) is a major cause of mortality in western society with around 37,000 new cases in the UK annually and a 5 year survival of ≈50% at presentation [1]. Despite advances in available techniques for treating metastases, the majority of patients remain incurable and existing adjuvant therapies such as chemotherapy are only of limited effectiveness. Understanding the molecular mechanisms underlying the metastatic process may allow us to identify those at greatest risk of recurrence and discover new tumour targets to prevent disease progression.

## 2. Matrix Composition and the Stroma

Those parameters thought to influence prognosis generally relate specifically to features of the carcinoma cells, with little attention being paid to ‘normal’ components of the tumour. However, it has become increasingly apparent that tumour stroma (including fibroblasts, inflammatory cells and endothelial cells) plays an important role in promoting tumour progression [2-5]. In many types of solid tumour SMA-positive myofibroblasts (peritumor fibroblasts, carcinoma-associated fibroblasts) are found within the stromal compartment [5]. Myofibroblasts are contractile, secretory cells, exerting tissue tension and producing extracellular matrix proteins and cytokines. Myofibroblasts have been reported to be associated with poor prognosis in several carcinoma types, including CRC [6-9].

Most commonly, myofibroblasts have been described as differentiating locally from fibroblasts [5]. However, it is now evident that a number of other cell types may undergo myofibroblastic transdifferentiation [5,10]: These include other locally-derived mesenchymal cells such as adipocytes, stellate cells and pericytes, as well as circulating mesenchymal stem cells and CD34-positive fibrocytes (which have CD14-positive monocytes as their precursor). What attracts circulating cells into the tumour remains to be fully elucidated, but the influx of fibrocytes in pulmonary fibrosis is mediated through the cytokine CXCL12 [11], and it is possible that similar chemo-attractant mechanisms play a role in generating CRC stroma [12]. Additionally, in recent years the concept of epithelial-to-mesenchymal (EMT) transition has received much attention, with suggestions that apparent stromal cells actually may be derived from epithelial tumour cells [13,14].

Several cytokines including TGF-β, PDGF, IL-4 and IGF-II have been reported to induce myofibroblastic differentiation [5,15]. TGF-β1 is a pleiotropic cytokine, which is over-expressed in many carcinomas, and may be pro-oncogenic [16]. A number of different activation mechanisms for TGF-β1 have been described including several classes of proteases, αvβ6 and αvβ8 integrins [17]. The relative role of each of these mechanisms in activating TGF-β1 *in vivo*, particularly in tumourigenesis, remains poorly understood [17]. However, TGF-β1-mediated Ras/Smad signaling [18-20] and contact with type I collagen mediated via α2β1 integrin [21] are believed to play a role in EMT in CRC, and αvβ6 has been suggested to be a marker of EMT in CRC and a prognostic factor for aggressive disease [22].

Other events are also required for myofibroblastic transdifferentiation to occur, including the presence of specialised extracellular matrix proteins such as the EDA- splice variant of fibronectin, and mechanical stress arising from integrin-dependent cell interactions with ECM proteins [23]. Osteopontin has also been reported to be required for myofibroblast transdifferentiation [24]. Osteopontin (OPN) positive macrophages in CRC may contribute to the development of myofibroblasic stroma [25], and are also believed to potentiate haematogenous spread of CRC by increasing microvascular density [26]. Reducing cellular tension and/or matrix compliance by inhibiting integrins αvβ3 and α5β1 [27,28] prevents myofibroblast development, as does inhibition of integrin-dependent FAK signaling [29]. Reactive oxygen species (ROS) promote myofibroblast transdifferentiation through both TGF-β1 -dependent and -independent mechanisms [5,17], an important consideration in patients who receive radiotherapy and chemotherapy. Much less is known about factors inhibiting myofibroblast differentiation. The inflammatory cytokines interferon γ and TNF-α have both been shown to inhibit transdifferentiation [5,30]. Webber and colleagues recently demonstrated a role for hyaluronan in maintaining a myofibroblastic phenotype by preventing apoptosis [31], and it is possible that the persistence of myofibroblasts in cancer stroma represents a defective apoptotic response. Interactions between the Hyaluronan receptors CD44 and RHAMM and hyaluronan synthases, play an important role in cancer cell extravasation and thereby metastatic spread [32]. In CRC high RHAMM receptor expression is a poor prognostic factor, postulated to be mediated via the Ras/MAPK pathway [33].

Myofibroblasts are associated with poor prognosis in several carcinoma types [34,35]. Tsujino and colleagues found that SMA expression was an independent marker of poor prognosis in colorectal cancer, and identified patients at high risk for disease recurrence [6]. It has also been suggested that the poorer prognosis of rectal carcinomas [36] is a result of extensive immature stroma associated with a down-regulated immune response [37]. Moreover, adenomas have been reported to contain increased myofibroblast numbers, suggesting that these cells may play a role in tumour initiation [38].

Myofibroblasts have now been shown to regulate a number of tumour-promoting functions, including angiogenesis, invasion and metastasis [5,10,39]. Myofibroblast density is usually greatest at the invasive front of the tumour, and several studies have shown that myofibroblasts promote CRC invasion secreting soluble factors such as HGF and SPARC [5,10]. Myofibroblasts also promote invasion by remodelling the extracellular matrix, and metalloproteinases (MMPs) and their inhibitors (TIMPs) produced by both cancer and stromal cells are known to play a role in altering the composition of the tumour microenvironment and are prognostic in CRC. High MMP-9 expression and low TIMP-2 and -3 expression confer a poor prognosis. Interestingly the distribution of MMP-9 varies throughout the tumour with lower stromal expression associated with the worst prognosis [40]. Myofibroblasts may also physically associate with tumour cells during the invasive process; Gaggioli and colleagues showed that fibroblasts lead collective tumour cell invasion a process dependent on the Rho-GTPase effector, Rock [41].

## 3. Tumour Microenvironment

**Figure 1.**
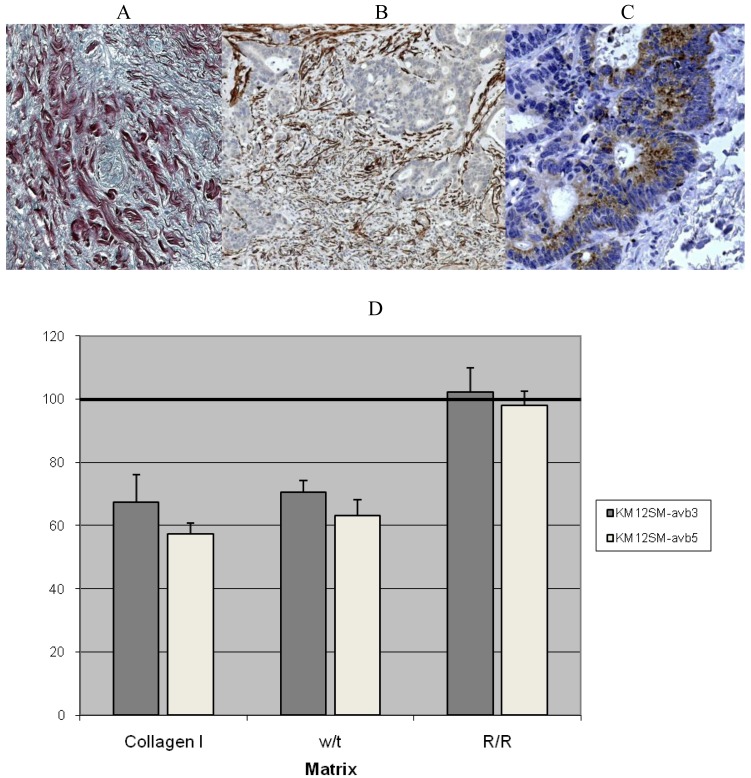
(A–C). CRC liver metastasis showing collagen expression (Sirius Red stain; A), myofibroblasts within the tumour deposit (SMA immunostain; B) and CRC αvβ5 integrin expression (C); (D) CRC proliferation was reduced when grown on r/r (protease-resistant) type 1 collagen compared to proprietary and wild type collagen I (data not shown). The addition of αvβ3 and αvβ5 neutralising antibodies effectively reduced the rate of proliferation for KM12SM (metastatic CRC) grown on proprietary and wild-type collagen I. In contrast these neutralising antibodies had no influence on KM12SM proliferation when grown on MMP resistant r/r collagen. These data suggest that matrix turnover plays an important role in regulating CRC growth mediated via αv integrin ligation. Means ± Confidence intervals, in comparison to IgG control (100%).

A myofibroblast-driven desmoplasic stromal reaction (DR), rich in fibrillar collagens (types I and III), is reported to be a poor prognostic indicator in primary CRC [42,43]. Metastatic deposits of CRC within the liver are also characterised by a pronounced DR associated with SMA-positive myofibroblasts derived from hepatic stellate cells [44]. We have shown previously the DR promotes growth and chemoresistance of CRC liver metastases (Figure 1). We found that up-regulation of CRC integrins αvβ5 and αvβ3 promoted cell survival through binding cryptic RGD binding sites on degraded type I collagen [45]. Several other studies have also postulated a key role for αv integrins in CRC [46,47] progression, and have shown that blocking αv integrins suppresses chemotherapy resistance [48] and decreases CRC invasiveness.

It is becoming apparent that myofibroblasts regulate numerous processes, which may be critical to CRC development and progression. In a recent study, Vermeulen and colleagues described a novel link between CRC stem cells and myofibroblasts, showing that myofibroblast-derived HGF activated CRC wnt signalling, and restored the stem cell phenotype in more differentiated cells [49]. These data suggest that the microenvironment is a critical regulator of the stem cell niche.

Interestingly, myofibroblasts may play a role in tumour immune evasion. The immune system plays a complex role in the development of CRC: The progression from adenoma to carcinoma is associated with a down-regulated Th1 response, resulting in reduced expression of cytokines IFN-gamma, TNF-alpha, IL-12 and IL-18 [50]. Although chronic inflammation, including inflammatory bowel disease, can be a precursor to CRC development, a pronounced anti-tumour CD3 +ve T-cell response is the best predictor of long-term survival for CRC patients [51], and the generation of antigen-specific cytotoxic T lymphocyte immunotherapy has been suggested as a possible treatment for several cancers [52]. Using the CT26 murine CRC model, Kraman and colleagues showed that fibroblast activation protein-α (FAP)-expressing tumour fibroblasts suppressed the tumour-directed adaptive immune response [53]. Moreover, depletion of these cells permitted immunological control of tumour growth. Targeting FAP-expressing stromal cells has been shown to inhibit CT26 tumor cell proliferation indirectly, decrease tumour myofibroblast content and blood vessel density [54]. These data suggest that targeting stromal cell-mediated modifications of the tumor microenvironment may be an effective approach to treating CRC.

**Figure 2.**
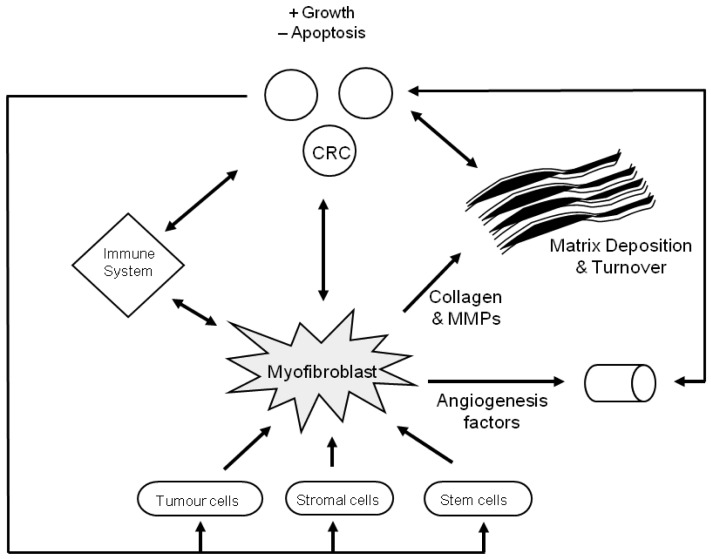
The role of the stroma in CRC development. Matrix deposition and turnover occurs simultaneously, as does the release of cytokines/factors which stimulate angiogenesis and alter immune function. This allows the colorectal cancer to grow and metastasise.

A possible model for stromal and CRC interactions is shown below (Figure 2).

## 4. Conclusions

It is increasingly clear that tumour stroma plays a crucial role in CRC development and progression. Understanding the role of the stromal cells and extracellular matrix will allow us to identify more precise prognostic markers and potentially devise new therapeutic options.
